# P30 A longitudinal perspective of antimicrobial resistance evolution within urinary tract infection-based clinical trials

**DOI:** 10.1093/jacamr/dlad077.034

**Published:** 2023-08-02

**Authors:** Phillip Aldridge, Aaron Tan, Alexandra Spencer, Maxime Vallée, Chris Harding, Judith Hall

**Affiliations:** Biosciences Institute, Newcastle University, Newcastle upon Tyne, UK; Biosciences Institute, Newcastle University, Newcastle upon Tyne, UK; Biosciences Institute, Newcastle University, Newcastle upon Tyne, UK; Biosciences Institute, Newcastle University, Newcastle upon Tyne, UK; Department of Urology, Poitiers University Hospital, Poitiers, France; Translational and Clinical Research Institute, Newcastle University, Newcastle upon Tyne, UK; Department of Urology, Freeman Hospital, Newcastle upon Tyne NHS Hospitals Foundation Trust, Newcastle upon Tyne, UK; Biosciences Institute, Newcastle University, Newcastle upon Tyne, UK

## Abstract

**Background:**

The mainstay treatments for urinary tract infections (UTIs) are nitrofurantoin and trimethoprim. The common route to acquire trimethoprim resistance (TriR) is via horizontal transfer of the trimethoprim-insensitive dihydrofolate reductase: *dfrA*. In contrast, nitrofurantoin resistance (NitR) requires the inactivation of two chromosomally encoded nitroreductases: *nfsA* and *nfsB*. The difference in antimicrobial resistance (AMR) evolution is reflected in community surveillance data: NitR incidence = ∼10% and for TriR > 30%. Longitudinal clinical UTI trials provide a unique opportunity to monitor in situ evolution of AMR and correlate its appearance to trial outcomes and the impact on uropathogens.

**Objectives:**

To explore the in situ AMR evolution and its impact in *Escherichia coli* isolates from patients participating in the clinical trials: AnTIC and ALTAR. AnTIC was an open label randomized trial assessing the efficacy of antibiotic prophylaxis use in clean intermittent self-catheterizing patients. ALTAR compared the efficacy of the non-antibiotic alternative methenamine hippurate to antibiotic prophylaxis to treat recurrent UTIs.

**Methods:**

The investigation of the evolution of nitrofurantoin and trimethoprim resistance in *E. coli* used general microbiology techniques, bioinformatic genome analysis and genetics to model known AMR mutations in susceptible *E. coli* strains.

**Results:**

Trimethoprim resistance amongst *E. coli* trial isolates was driven by 14 out of ∼45 known *dfrA* allelic variants; the most common being *dfrA1* and *dfrA17*. Growth analysis identified an allelic bias when strains were exposed to sub-MIC concentrations of trimethoprim. Growth rate analysis identified a 2%–10% slower doubling time for nitrofurantoin-resistant strains: NitS: 20.8 ± 0.7 min compared to NitR: 23 ± 0.8 min. Statistically, these data suggested no fitness advantage of evolved strains compared to the sensitive predecessor (*P* value = 0.13). Genetic manipulation of *E. coli* to mimic NitR evolution, however, supported a selective advantage.

**Conclusions:**

Longitudinal sampling during antibiotic based clinical trials has provided a new perspective to the response of *E. coli* to UTI-related antibiotics. Correlation of nitrofurantoin response to pharmacokinetic data suggests that the low incidence of *E. coli* NitR is driven by selection not fitness. However, fitness correlates to *dfrA* allele carriage and could potentially be exploited to benefit the use of trimethoprim if other antibiotics are impeded by AMR.

